# White matter hyperintensities and late-life cognition: an analysis of moderation effects of mild behavioral impairment

**DOI:** 10.3389/fnagi.2025.1675465

**Published:** 2026-01-20

**Authors:** Jiayu Duan, Ningqun Wang, Shixin Li, Yefei Wang, Jiale Song, Xiaoshan Li, Jingrui Guo, Junya Liao, Yihan Wang, Ying Zhang, Yunling Zhang, Xianglan Jin

**Affiliations:** 1 Beijing University of Chinese Medicine, Beijing, China; 2 Department of Neurology, Dongfang Hospital, Beijing University of Chinese Medicine, Beijing, China; 3 Department of Neurology, Xuanwu Hospital of Capital Medical University, Beijing, China; 4 Department of Neurology, Xiyuan Hospital, China Academy of Chinese Medical Sciences, Beijing, China

**Keywords:** arteriosclerotic cerebral small vessel disease, mild behavioral impairment, moderation effect, screen-positive cognitive impairment, white matter hyperintensities

## Abstract

**Background:**

Mild behavioral impairment (MBI) is recognized as a potential early marker for dementia and may be indicative of the increased risk in the predementia stage. This study investigated whether MBI could moderate the impact of white matter hyperintensities (WMHs) on late-life cognitive function in patients with arteriosclerotic cerebral small vessel disease (aCSVD).

**Methods:**

Patients were categorized into two groups based on Fazekas scores: low WMH burden (LWMH, scores 1–2; *n* = 119) and high WMH burden (HWMH, scores 3–6; *n* = 110). Logistic regression analysis was used to assess the associations among WMH, MBI, and screen-positive cognitive impairment. Moderation analysis was conducted to evaluate whether the presence of MBI could influence the relationship between WMH and cognitive outcomes.

**Results:**

WMH was associated with MBI (*p* < 0.001) and screen-positive cognitive impairment (MoCA: *p* = 0.003; MMSE: *p* = 0.025), and MBI was correlated with screen-positive cognitive impairment (*p* = 0.010 and 0.007, respectively). HWMH was independently correlated with screen-positive cognitive impairment and MBI (*p* = 0.001; *p* < 0.001; *p* = 0.031). Compared with cases in the non-MBI group, those in the MBI group had more WMHs in the occipital lobes (*p* = 0.016) and subcortical structures (*p* = 0.042). Furthermore, MBI significantly moderated the association between WMH and screen-positive cognitive impairment (β = −0.10, *p* < 0.014; β = −0.06, *p* < 0.041).

**Conclusion:**

The presence of MBI may exacerbate the cognitive decline associated with WMH in patients with aCSVD. These findings highlight the importance of early identification and monitoring of MBI in cases with WMH to better assess dementia risk and to develop interventional strategies.

## 1 Introduction

Cerebral small vessel disease (CSVD) is not only a common chronic vascular condition (Chojdak-Łukasiewicz et al., 2021), but also a significant contributor to vascular cognitive impairment (VCI), which is frequently identified through positive findings on vascular screening tests, affecting up to 67% of cases (Peng and Shao, 2019). Arteriosclerotic CSVD (aCSVD) is the most frequent subtype of CSVD. It typically has an insidious onset and gradually progresses, with a subset of patients eventually developing screen-positive cognitive impairment (CI). The prevalence of CI associated with aCSVD is rising; however, its early clinical manifestations are typically subtle and easily overlooked by patients (Wei et al., 2024). Early identification of aCSVD-related cognitive decline is crucial, as timely intervention may delay the progression to dementia and mitigate the burden on families and society (Barucci et al., 2024; Zhou et al., 2018).

Early identification of aCSVD symptoms is critical for the timely clinical diagnosis. The pattern of cognitive changes in the early stage of aCSVD overlaps with that of screen-positive mild cognitive impairment (MCI; Peng and Shao, 2019). A clinical staging diagnosis rather than a distinct etiological entity. Additionally, some patients with aCSVD may present with neurobehavioral symptoms. Mild behavioral impairment (MBI), defined as persistent, late-onset behavioral changes, has emerged as a potential prodromal marker of dementia (Ismail et al., 2016). A retrospective study clinically reclassified patients as psychiatric or MBI, in which those in the MBI group exhibited a significantly higher hazard ratio for developing dementia (Matsuoka et al., 2019). Importantly, MBI has emerged as an independent, non-cognitive marker of dementia. It is reported to occurs in 35%−85% of cases with MCI (Monastero et al., 2009), and its presence significantly increases the risk of progression to dementia by up to 25% (Ismail et al., 2017). Incorporating MBI into risk assessments enhances the clinical prognostic evaluation of patients with MCI (McGirr et al., 2022). Although prior studies have linked MBI to both white matter hyperintensities (WMH) and screen-positive CI, its role as a moderator in the relationship between WMH and cognition has not been previously investigated.

Early diagnosis of aCSVD requires neuroimaging data. WMHs are a common imaging manifestation of aCSVD, and numerous studies have demonstrated an association between WMHs and screen-positive CI in aCSVD. The severity of screen-positive CI is generally positively correlated with the extent of WMHs, primarily influencing executive function and information processing speed (Xia et al., 2020; Wang et al., 2020; Jokinen et al., 2020). However, it is noteworthy that the presence of WMHs in patients with aCSVD does not invariably lead to screen-positive CI, and the progression of screen-positive CI can be influenced by several comorbid factors (Chen et al., 2021). Although MBI is a precursor symptom to the diagnosis of clinical dementia, few studies have investigated whether comorbid MBI may influence cognitive function in patients with aCSVD and WMHs.

Screen-positive CI is increasingly recognized as a major clinical manifestation of aCSVD associated with WMHs. Early assessment of MBI may facilitate the identification of dementia at preclinical or prodromal stages. Therefore, investigating the relationship among WMHs, MBI, and cognitive function in aCSVD is essential. This study aimed to examine the association between WMHs and screen-positive CI, and to further explore the moderating role of MBI in the relationship between WMHs and screen-positive CI among patients with aCSVD.

## 2 Methods

### 2.1 Participants

A cross-sectional research was conducted on 229 consecutive patients with aCSVD who were admitted to the outpatient and inpatient neurology departments of Dongfang Hospital Affiliated to Beijing University of Chinese Medicine (Beijing, China) from July 2019 to May 2021. The inclusion criteria were as follows: (1) age between 50 and 80 years; (2) magnetic resonance imaging (MRI) showing WMHs of presumed vascular origin; (3) cognitive screening criteria: Montreal Cognitive Assessment (MoCA) score <26 (with an additional point for participants with less than 9 years of education), or Mini-Mental State Examination (MMSE) score between 19 and 26 (17–26 for individuals with no formal education, with 24 as the cutoff for those with no formal education). MoCA and MMSE were administered independently, and participants also had a Clinical Dementia Rating (CDR) score of ≤1; and (4) adequate visual, auditory, and language abilities, or the ability to complete neuropsychological assessments with correction. Exclusion criteria were as follows: (1) diagnosis of sporadic or hereditary cerebral amyloid angiopathy, hereditary small vessel disease due to non-amyloid vascular degeneration, inflammatory or immune-mediated small vessel disease, venous collagen disease, or other non-arteriosclerotic small vessel diseases; (2) white matter lesions attributed to cortical or watershed infarcts, cerebral hemorrhage, hydrocephalus, or other conditions, such as multiple sclerosis, carbon monoxide poisoning, hypoxic encephalopathy, or hypoglycemia; (3) mild Alzheimer's disease (AD), defined by the 2011 National Institute on Aging-Alzheimer's Association (NIA-AA) core clinical criteria for probable AD dementia (McKhann et al., 2011), or MRI evidence of medial temporal lobe atrophy (medial temporal lobe atrophy scale ≥2; Ferreira et al., 2015); (4) screen-positive CI due to other clearly identifiable causes; (5) diagnosis of moderate-to-severe depression or other psychiatric/behavioral disorders; (6) contraindications to undergoing MRI; and (7) refusal to provide a blood sample.

This research is a sub-study of the China Cognition and Aging Study, which was conducted by Dongfang Hospital, and it was approved by the Ethics Committee of Xuanwu Hospital, Capital Medical University (Approval No. 2017004). All study procedures were pre-specified and remained unchanged throughout the study period. The research was conducted in accordance with the guidelines and regulations of the Ethics Committee of Xuanwu Hospital, concentrating on protecting all participants' rights and wellbeing. During the recruitment process, all subjects provided written informed consent. The research was carried out based on the Strengthening the Reporting of Observational Studies in Epidemiology statement (von Elm et al., 2007).

### 2.2 Cognitive assessment

All participants underwent cognitive screening via the Chinese versions of the MoCA and MMSE in the present study (Lu et al., 2011; Katzman et al., 1988). The MoCA and the MMSE were administered by two qualified researchers. The MoCA evaluates multiple cognitive domains, including visuospatial/executive function, naming, attention, language, abstraction, memory, and orientation. The MMSE assesses orientation, registration, attention and calculation, recall, language, and praxis. Cognitive function was screened using the following score thresholds for potential screen-positive CI: MoCA score between 19 and 26 (17 for individuals with no formal education), with an additional point granted for participants with fewer than 9 years of education, or MMSE score ≤26 (24 for individuals with no formal education). It should be noted that these thresholds are utilized only for screening purposes and do not serve as a formal diagnosis of MCI, mild dementia, or other cognitive disorders. A comprehensive neurological and neuropsychological evaluation is required for the establishment of a definitive diagnosis (Tian et al., 2021).

### 2.3 MBI checklist

MBI was evaluated via the MBI Checklist (MBI-C). It is a publicly accessible, verified tool, available at MBItest.org, which was designed exclusively to identify MBI instances (Ismail et al., 2017; Lin et al., 2023). The MBI-C consists of 34 items divided into five domains: (1) impaired drive/motivation, assessed by 6 questions covering behavioral, cognitive, and emotional apathy; (2) affective/emotional dysregulation, assessed by 6 questions addressing low mood, guilt, hopelessness, anhedonia, worry, and panic; (3) impulse dyscontrol, assessed by 12 questions evaluating aggression, recklessness, impulsivity, agitation, abnormal reward, and reinforcement; (4) social inappropriateness, assessed by five questions on tact, sensitivity, and empathy; and (5) abnormal thoughts/perception, assessed by five questions on grandiosity, suspiciousness, and auditory and visual hallucinations. Symptoms must persist for a minimum of 6 months, representing a meaningful variation from baseline. Items are rated as “yes” or “no.” If an item is rated as “yes,” the severity of the item is rated as 1 (mild), 2 (moderate), or 3 (severe) (Ismail et al., 2017). Patients were divided into the MBI group and the non-MBI group using a cutoff point of MBI-C score ≥6.5 (Mallo et al., 2018).

### 2.4 Brain MRI and assessment of white matter lesions

T1 and T2 FLAIR images were acquired using a GE DISCOVERY MR750 3.0T MRI scanner at the Imaging Centre of Dongfang Hospital, Beijing University of Chinese Medicine. Additionally, each subject underwent routine axial T1- and T2-weighted sequences. On MR images, WMHs appear as abnormal signals of varying sizes within the white matter: hyperintense on T2-weighted and FLAIR sequences, and iso- or hypointense on T1-weighted sequences.

The location of WMHs was evaluated using Standards for Reporting Vascular Changes on Neuroimaging 2 criteria (Duering et al., 2023) and determined according to the presence or absence of lesions. WMHs were assessed separately in the frontal, temporal, parietal, and occipital lobes, brainstem, subcortical structures, and juxtacortical regions.

**Figure 1:**
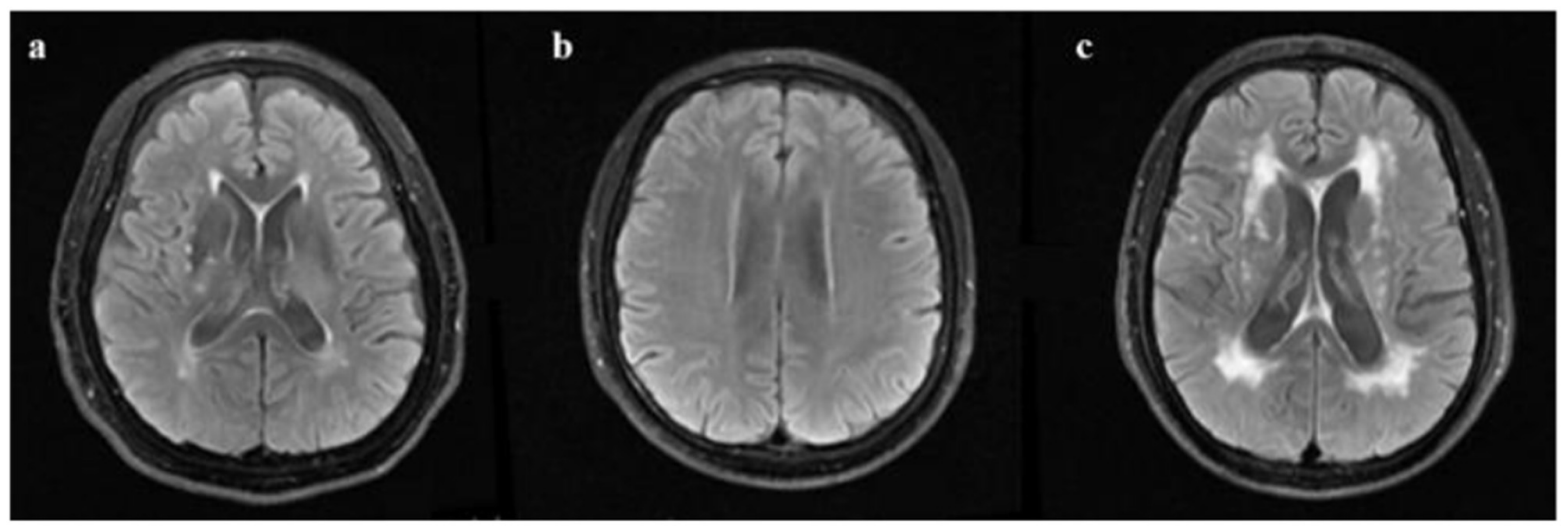
Fazekas scale criteria of periventricular white matter hyperintensities. **(a)** “Caps” or pencil-thin lining; **(b)** smooth “halo”; **(c)** irregular periventricular hyperintensity extending into the deep white matter (examples of T2 FLAIR from patients with aCSVD).

**Figure 2:**
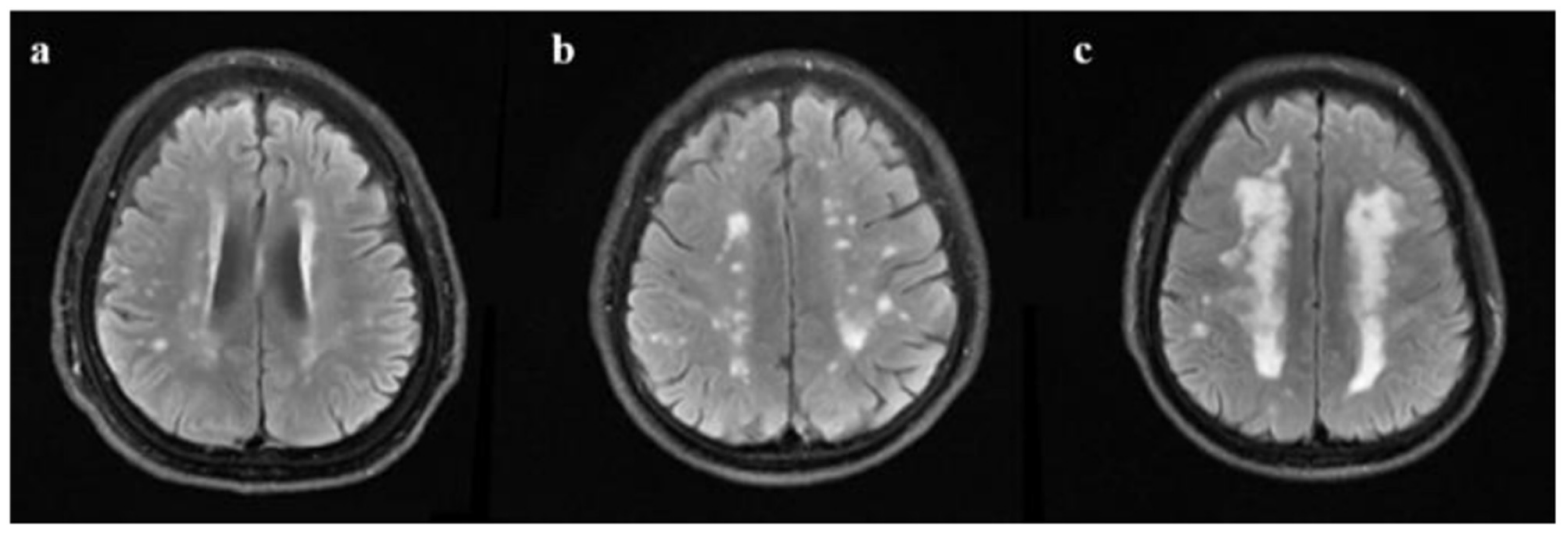
Fazekas scale scores of deep white matter hyperintensities. **(a)** punctate foci; **(b)** beginning confluence of foci; **(c)** large confluent areas (instances of T2 FLAIR from patients with aCSVD).

The Fazekas scores were calculated, according to the guidelines using FLAIR MRI sequences (Fazekas et al., 1987), to assess the extent of leukoaraiosis. In brief, periventricular and deep WMHs were assessed separately, and their scores were combined to obtain a total score ranging from 0 to 6. Based on the Fazekas scores, patients were categorized into two groups: lower WMH (LWMH) with scores of 1–2, and higher WMH (HWMH) with scores of 3–6. MRI manifestations and the Fazekas scores were jointly assessed by a neurologist and a radiologist. Any differences in the interpretation of the images were resolved through discussion, yielding unified results (Figures 1, 2).

### 2.5 Clinical and laboratory indices

General demographic data (age, gender, body mass index (BMI), and educational level), medical history (hypertension, diabetes, and hyperlipidemia), and laboratory indices (fasting blood glucose, total cholesterol, and homocysteine) were collected.

### 2.6 Statistical analysis

The data's normality was examined via the Shapiro–Wilk test. The normally distributed variables were presented as mean ± standard deviation, and the median (IQR) was used to represent variables with an abnormal distribution, along with the application of frequency (%) to express count data. Parametric tests were applied to normally distributed variables, while nonparametric tests were used for variables with abnormal distributions. Spearman correlation analysis examined the relationships among WMHs, MBI, and cognitive performance. Demographic and clinical characteristics, cognitive function, and MBI were compared between the LWMH and HWMH groups, and WMH location was compared between the MBI and non-MBI groups using independent-sample *t*-test, Chi-square test, and Mann-Whitney *U*-test. Logistic regression analysis was performed to assess the associations of WMH with MBI and cognitive function, adjusting for potential confounders. The covariates included age, gender, and educational level, as these factors are well-documented to have significant impacts on cognitive performance and MBI prevalence in older adults (Lu et al., 2011; Katzman et al., 1988; Mallo et al., 2018). Stratified analysis was not selected due to the need to retain statistical power for moderation testing (which requires sufficient sample size across all levels of the moderator and independent variable) and the conceptual goal of adjusting for these confounders rather than testing their interactive effects with WMH or MBI. *Z*-scores were calculated to represent the sub terms of MoCA, MMSE, and MBI-C, in order to eliminate differences in score inconsistency. The results were expressed as adjusted odds ratios (ORs) and 95% confidence intervals (CIs).

Moderation analyses were performed to investigate the moderating effect of MBI on the relationship between WMH and cognition. Firstly, all variables were normalized to a 0–1 scale using min–max normalization (Liu et al., 2023) to facilitate comparison and interpretation. Secondly, Model 1 of the Process Macro (Hayes, 2015) was employed to test moderation effects, and age, gender, and educational level were included as covariates to control for their potential influence. Finally, 95% CIs were estimated using bootstrapping with 5,000 samples. Given the number of cognitive domains (*n* = 7) and MBI subdomains (*n* = 5) analyzed, we considered applying false discovery rate (FDR) correction to account for multiple comparisons. Given the exploratory nature of this study and the relatively high inter-correlation among certain cognitive domains (e.g., attention and executive function), we decided to report uncorrected *p*-values. However, we acknowledge the potential for Type I error, and therefore, the results, especially those exhibiting borderline significance (e.g., *p*-values between 0.01 and 0.05), should be interpreted with caution. Future confirmatory studies should consider applying stricter correction methods, such as Bonferroni or FDR, to enhance statistical rigor and reduce the likelihood of false-positive findings.

Analyses were performed with PASW 26.0 software, and the figures were visualized using GraphPad Prism 9.2.0 software. The significance level was set at *p* < 0.05.

## 3 Results

### 3.1 Demographic characteristics

Table 1 summarizes the detailed features of the research population. Among 229 patients with aCSVD, 87 were women (37.99%), 119 had LWMH (51.97%), and 110 had HWMH (48.03%). Participants aged 50–80 years old, with an average age of 65.64 ± 7.34 years old. Significant differences were found in age between the two groups. Participants were older in the HWMH group (*p* < 0.001) vs. those in the LWMH group.

**Table 1:** Characteristics of the study population.

**Characteristics**	**LWMH (*n* = 119)**	**HWMH (*n* = 110)**	***p*-value**
Age, mean ± SD (range), years	63.88 ± 7.10 (62.59–65.17)	67.55 ± 7.15 (66.19–68.90)	<0.001*
Women (*n*, %)	52 (43.70%)	35 (31.82%)	0.064
BMI, mean ± SD (range), kg/m^2^	24.59 ± 2.95 (24.06–25.13)	24.78 ± 3.00 (24.21–25.35)	0.576
Education, median (IQR), years	12.00 (9.00–12.00)	9.50 (9.00–15.00)	0.425
**Medical history**, ***n*** **(%)**			
Hypertension	81 (68.07%)	81 (73.64%)	0.355
Diabetes	47 (39.50%)	46 (41.82%)	0.721
Hypercholesterolaemia	63 (52.94%)	59 (53.64%)	0.916
Smoking	56 (47.06%)	58 (52.73%)	0.391
Drinking	52 (43.70%)	45 (40.91%)	0.670
**Blood test at admission, median (IQR)**			
Fasting blood glucose, mmol/L	5.87 (5.19–6.96)	6.33 (5.11–8.02)	0.407
Total cholesterol, mmol/L	4.13 (3.47–4.91)	3.93 (3.30–4.65)	0.102
Homocysteine, μmol/L	13.55 (10.58–16.45)	13.30 (10.98–17.15)	0.650

LWMH, lower white matter hyperintensities; HWMH, higher white matter hyperintensities; BMI, body mass index.

* p < 0.05.

### 3.2 Correlations among WMH, MBI, and cognitive function in aCSVD

**Figure 3:**
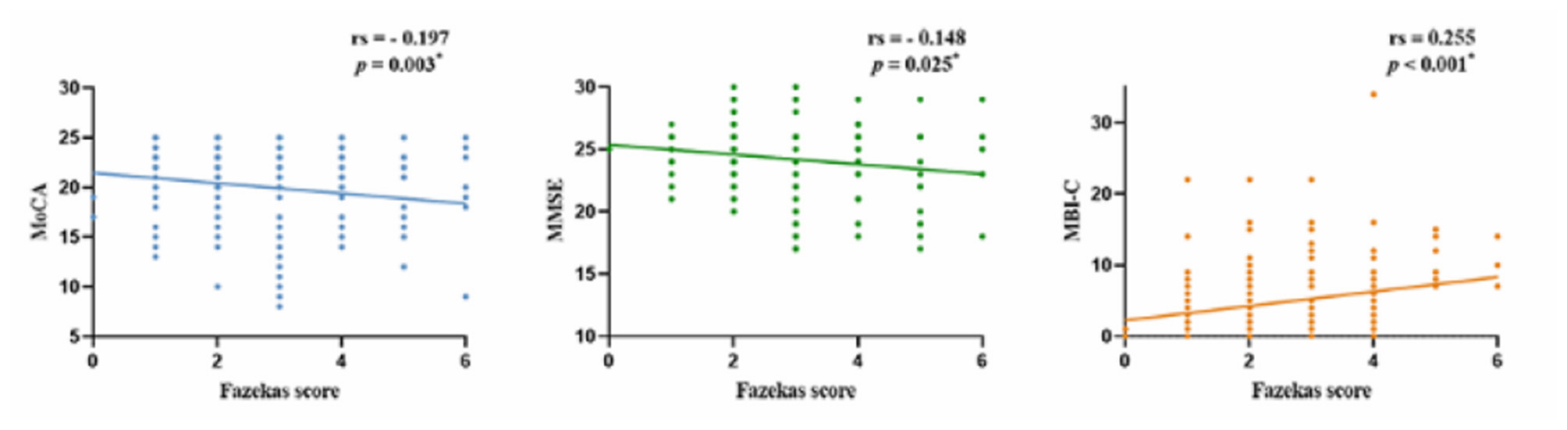
Correlation of WMHs with cognitive function and MBI. WMHs, white matter hyperintensities; MBI, mild behavioral impairment; MBI-C, MBI-checklist; MoCA, Montreal Cognitive Assessment; MMSE, Mini-Mental State Examination; *p < 0.05.

**Figure 4:**
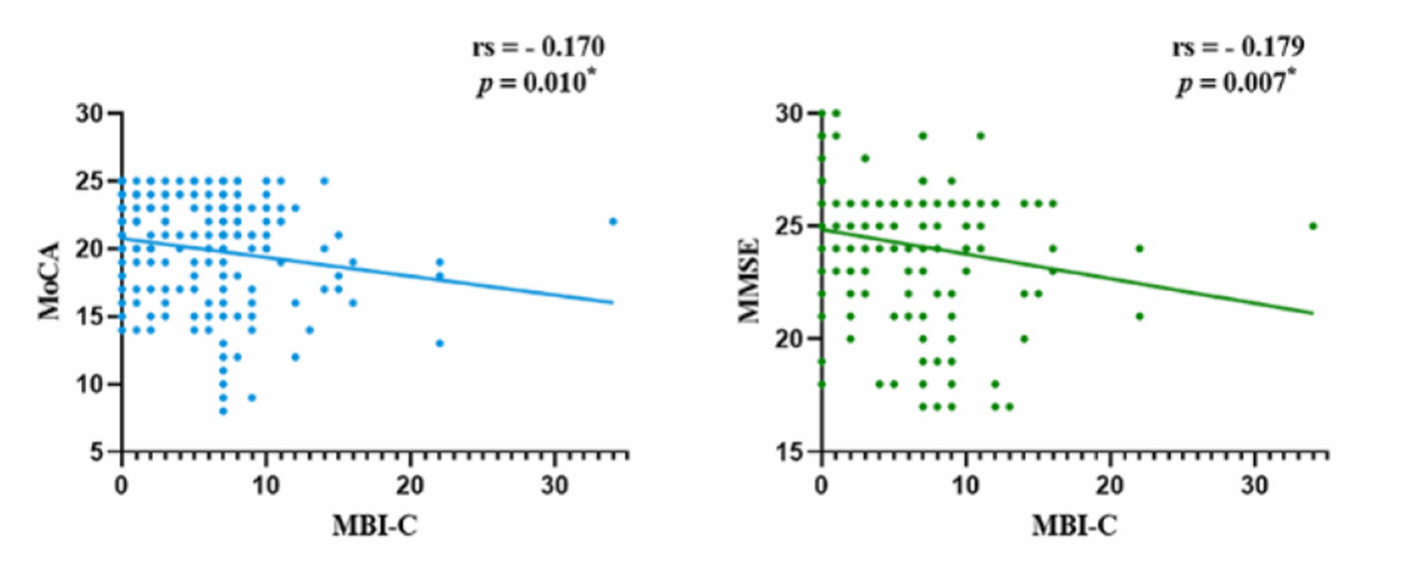
Correlation between MBI and cognitive function. MBI, mild behavioral impairment; MoCA, Montreal Cognitive Assessment; MMSE, Mini-Mental State Examination; *p < 0.05.

The Fazekas scores were negatively correlated with the MoCA and MMSE scores (rMoCA = −0.197, *p* = 0.003; rMMSE = −0.148, *p* = 0.025), while positively correlated with the MBI-C scores (rMBI-C = 0.255, *p* < 0.001). The MBI-C scores were negatively correlated with the MoCA and MMSE scores (rMoCA = −0.170, *p* = 0.010; rMMSE = −0.179, *p* = 0.007; Figures 3, 4).

### 3.3 WMHs could affect cognitive function and MBI in aCSVD patients

Participants in the HWMH group had worse cognitive function than those in the LWMH group, as evidenced by attention, executive function, memory, and language capabilities. Following adjustment for age, gender, and educational level (VIF < 10), the overall risk of CI (as reflected by MoCA and MMSE scores), especially in attention, executive function, memory, and orientation, was higher in the HWMH group than that in the LWMH group (OR = 0.87, 95% CIs: 0.80–0.94, *p* = 0.001; OR = 0.77, 95% CIs: 0.68–0.87, *p* < 0.001; OR = 0.57, 95% CIs: 0.42–0.79, *p* = 0.001; OR = 0.67, 95% CIs: 0.50–0.90, *p* = 0.008; OR = 0.70, 95% CIs: 0.52–0.93, *p* = 0.016; OR = 0.65, 95% CIs: 0.49–0.86, *p* = 0.003; OR = 0.69, 95% CIs: 0.51–0.94, *p* = 0.019; Table 2).

**Table 2:** Differences in cognitive function between LWMH and HWMH.

**Cognitive Domains/Scales**	**LWMH**	**HWMH**	***p*-value**	**OR (95% CIs), *p*-value**
MoCA	22.00 (19.00–24.00)	20.00 (16.00–23.00)	<0.001*	0.87 (0.80, 0.94), 0.001*
Visuospatial/executive	0.22 (−0.50 to 0.94)	0.22 (−0.50 to 0.94)	0.116	0.85 (0.63, 1.13), 0.249
Naming	0.43 (0.43–0.43)	0.43 (0.43–0.43)	0.122	1.08 (0.82, 1.42), 0.580
Attention	0.18 (−0.63 to 0.98)	0.18 (−0.63 to 0.98)	<0.001*	0.57 (0.42, 0.79), 0.001*
Language	0.44 (−0.72 to 0.44)	0.44 (−0.72 to 0.44)	0.032*	0.80 (0.60, 1.05), 0.109
Abstraction	−0.05 (−0.45 to 1.25)	−0.05 (−1.34 to 1.25)	0.180	0.86 (0.65, 1.15), 0.307
Memory	0.16 (−0.58 to 0.91)	−0.58 (−1.33 to 0.91)	<0.001*	0.65 (0.49, 0.86), 0.003*
Orientation	0.69 (−0.38 to 0.69)	0.69 (−0.38 to 0.69)	0.601	0.89 (0.67, 1.18), 0.407
MMSE	26.00 (24.00–26.00)	24.00 (21.00–26.00)	0.002*	0.77 (0.68, 0.87), <0.001*
Orientation	0.19 (−0.57 to 0.95)	0.19 (−0.57 to 0.95)	0.110	0.69 (0.51, 0.94), 0.019*
Registration	0.29 (0.29–0.29)	0.29 (0.29–0.29)	0.465	1.20 (0.91, 1.60), 0.198
Attention and calculation	0.42 (−0.91 to 1.09)	−0.24 (−0.91 to 1.09)	0.018*	0.67 (0.50, 0.90), 0.008*
Recall	0.44 (−0.67 to 0.44)	−0.67 (−0.67 to 0.44)	0.084	0.80 (0.60, 1.04), 0.098
Language and praxis	0.25 (−0.53 to 1.02)	0.25 (−0.53 to 1.02)	0.063	0.70 (0.52, 0.93), 0.016*

LWMH, lower white matter hyperintensities; HWMH, higher white matter hyperintensities; MoCA, Montreal Cognitive Assessment; MMSE, Mini-Mental State Examination.

* p < 0.05.

The HWMH group also had worse MBI than the LWMH group, as evidenced by impaired drive/motivation and affective/emotional dysregulation. Following adjustment for age, gender, and educational level (VIF < 10), the risk of MBI, especially in impaired drive/motivation and affective/emotional dysregulation, was higher in the HWMH group than that in the LWMH group (OR = 1.07, 95% CIs: 1.01–1.13, *p* = 0.031; OR = 1.51, 95% CIs: 1.11–2.06, *p* = 0.008; OR = 1.55, 95% CIs: 1.16–2.08, *p* = 0.003; Table 3).

**Table 3:** Differences in MBI between LWMH and HWMH.

**MBI Domains/Scale**	**LWMH**	**HWMH**	***p*-value**	**OR (95% CIs), *p*-value**
MBI-C	3.00 (1.00–7.00)	7.00 (0.75–8.00)	0.016*	1.07 (1.01, 1.14), 0.031*
Impaired drive/motivation	−0.37 (−0.87 to 0.13)	0.13 (−0.87 to 0.63)	<0.001*	1.51 (1.11, 2.06), 0.008*
Affective/emotional dysregulation	−0.29 (−0.96 to 0.38)	0.38 (−0.96 to 1.05)	0.001*	1.55 (1.16, 2.08), 0.003*
Impulse dyscontrol	−0.69 (−0.69 to 0.68)	−0.69 (−0.69 to 0.68)	0.830	1.02 (0.78, 1.33), 0.898
Social inappropriateness	−0.50 (−0.50 to −0.50)	−0.50 (−0.50 to 0.61)	0.569	1.04 (0.79, 1.37), 0.768
Abnormal thoughts/perception	−0.39 (−0.39 to −0.39)	−0.39 (−0.39 to −0.39)	0.528	1.01 (0.77, 1.32), 0.966

MBI, mild behavioral impairment; LWMH, lower white matter hyperintensities; HWMH, higher white matter hyperintensities; MBI-C, MBI-Checklist.

* p < 0.05.

**Figure 5:**
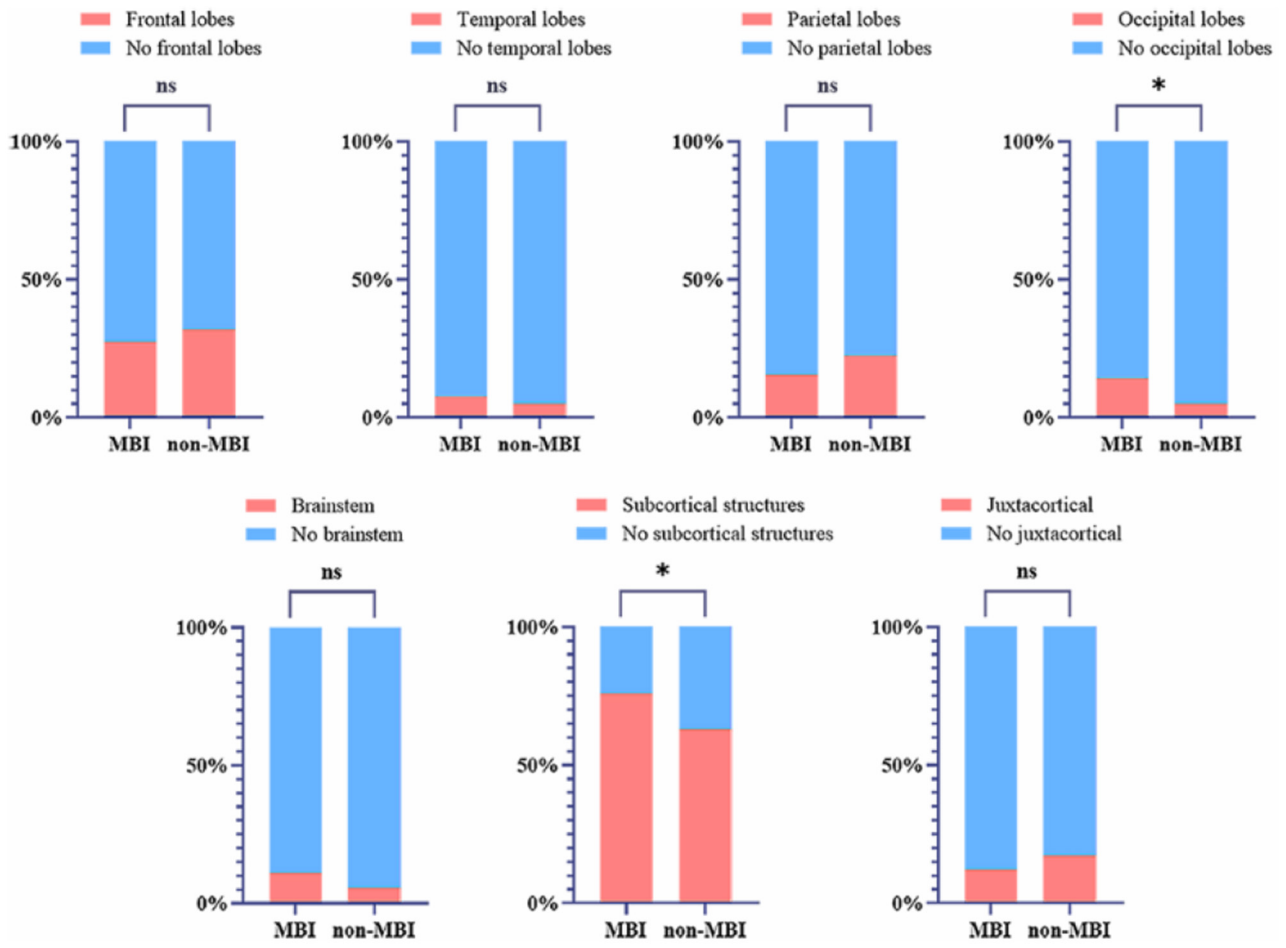
Differences in locations of WMHs between MBI and non-MBI groups. WMHs, white matter hyperintensities; MBI, mild behavioral impairment; *p < 0.05.

Patients were then divided into the MBI group (*n* = 92) and non-MBI group (*n* = 137) based on the MBI-C cutoff score of ≥6.5. To explore the regional distribution of WMHs associated with MBI, WMH locations were compared between the two groups. As illustrated in Figure 5, the MBI group had a significantly higher proportion of WMH involvement in the occipital lobes (*p* = 0.016) and subcortical structures (e.g., basal ganglia, thalamus; *p* = 0.042) compared with the non-MBI group. In contrast, no significant differences were found in WMH involvement in the frontal lobes, temporal lobes, parietal lobes, brainstem, or juxtacortical regions between the two groups (all *p* > 0.05). These findings demonstrate that WMHs in the occipital lobes and subcortical structures may be particularly associated with the presence of MBI in aCSVD patients.

### 3.4 Moderation analysis of MBI

**Figure 6:**
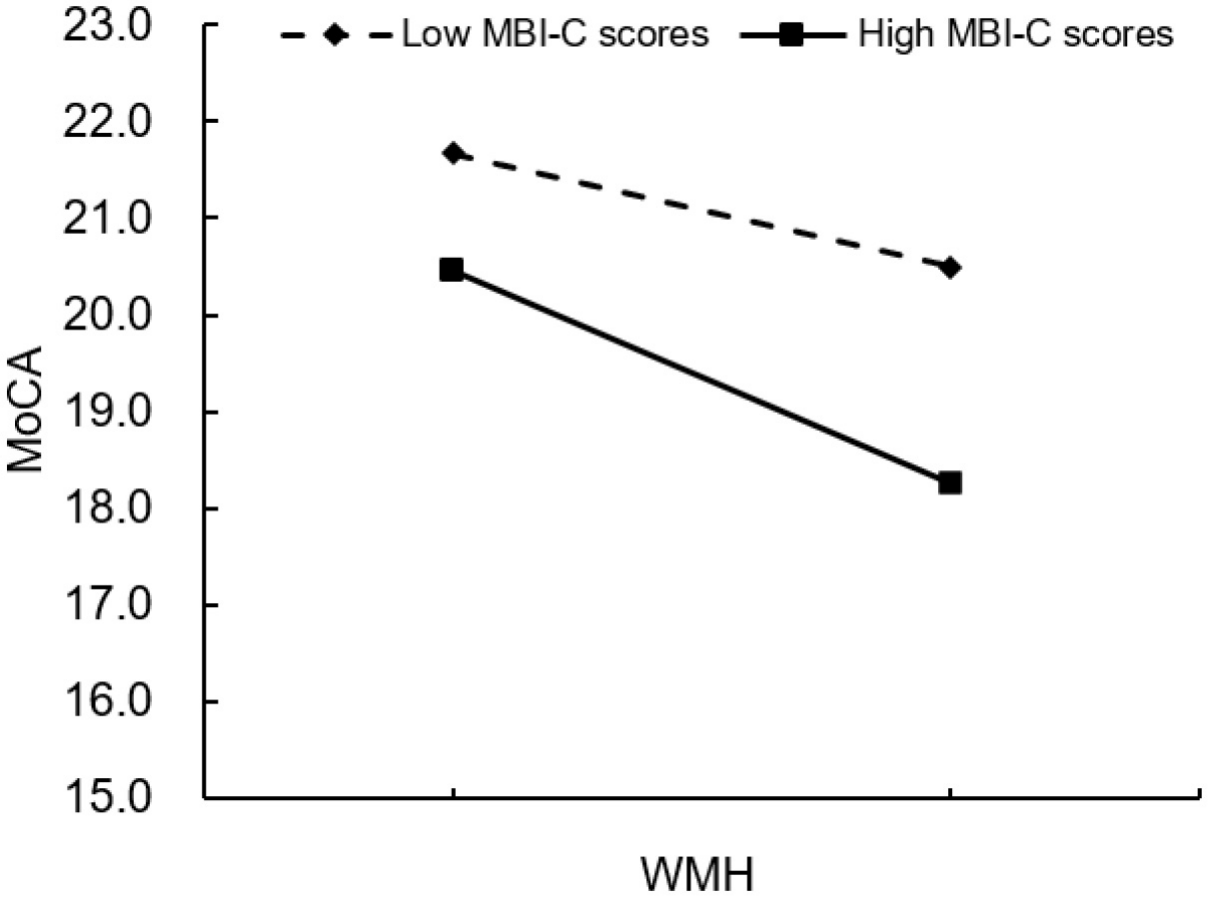
Plot of interaction between WMH and MBI on screen-positive cognitive impairment (outcome variable: MoCA). WMH, white matter hyperintensities; MBI, mild behavioral impairment; MoCA, Montreal Cognitive Assessment; *p < 0.05.

**Figure 7:**
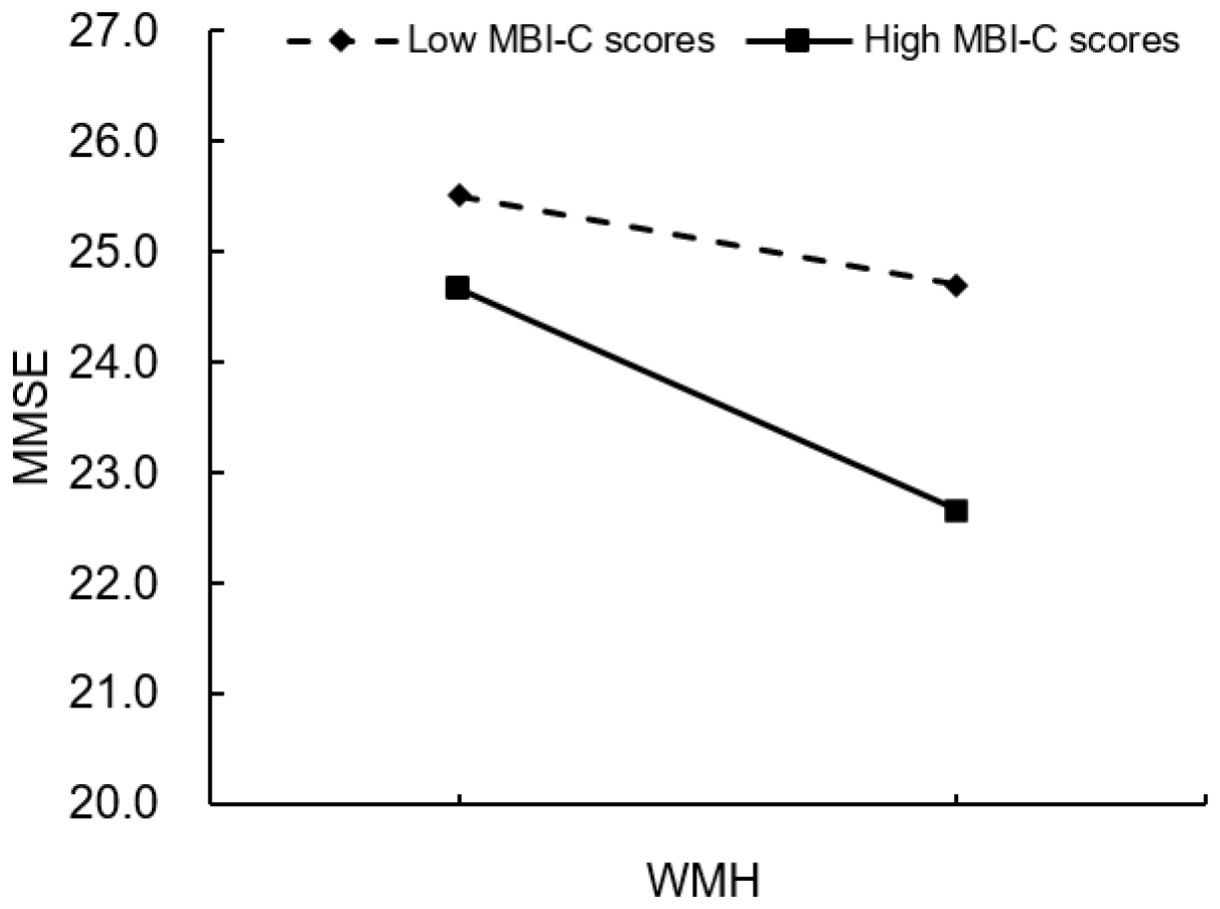
Plot of interaction between WMH and MBI on screen-positive cognitive impairment (outcome variable: MMSE). WMHs, white matter hyperintensities; MBI, mild behavioral impairment; MMSE, Mini-Mental State Examination; *p < 0.05.

Table 4 presents the results of moderation analysis. After controlling for age, gender, and educational level, the Fazekas scores were negatively correlated with the MoCA and MMSE scores (β = −0.43, *p* < 0.030; β = −0.37, *p* < 0.010), the MBI-C scores were also negatively correlated with the MoCA and MMSE scores (β = –0.12, *p* < 0.017; β = –0.09, *p* < 0.012), and the correlation of Fazekas scores with MBI-C scores exhibited a significant effect on the MoCA and MMSE scores (β = **–**0.10, *p* < 0.014; β = –0.06, *p* < 0.041). The effect of WMH on the MoCA and MMSE scores at different MBI levels is depicted in Figures 6, 7.

**Table 4:** Moderating role of MBI in the association between WMH and screen-positive cognitive impairment.

**Variables**	**β**	**SE**	***t***	***p***	**95% CIs**
**Moderating variable models (outcome variable: MoCA)**					
Constant	14.27	2.35	6.08	<0.001*	9.65, 18.89
Fazekas scores	−0.43	0.20	−2.18	0.030*	−0.83, −0.04
MBI-C	−0.12	0.05	−2.41	0.017*	−0.23, −0.02
Fazekas scores × MBI-C	−0.10	0.04	−2.47	0.014*	−0.02, −0.18
**Moderating variable models (outcome variable: MMSE)**					
Constant	17.33	1.69	10.27	<0.001*	14.01, 20.66
Fazekas scores	−0.37	0.14	−2.59	0.010*	−0.66, −0.09
MBI-C	−0.09	0.04	−2.54	0.012*	−0.17, −0.02
Fazekas scores × MBI-C	−0.06	0.03	−2.05	0.041*	−0.01, −0.12

* *p* < 0.05.

## 4 Discussion

In this cross-sectional study, after adjusting for age, gender, and educational level, a HWMH burden was associated with more severe screen-positive CI and MBI compared with a LWMH burden. Compared to aCSVD patients without MBI, those with MBI exhibited greater WMH involvement in the occipital lobes and subcortical structures. Furthermore, MBI moderated the association between WMHs and screen-positive CI in aCSVD patients. Early recognition of aCSVD symptoms is critical for timely clinical diagnosis. CSVD accounts for approximately 45% of dementia cases (Gorelick et al., 2011), and screen-positive CI and dementia have become central focuses of aCSVD research. Identifying preclinical predictors of dementia remains a key objective. While prior studies have primarily concentrated on the relationship between MCI and dementia, recent research suggested that MBI could also increase dementia risk and accelerate its onset (Jiang et al., 2022). The present study included aCSVD patients with cognitive screening results consistent with MCI and mild dementia [defined by MoCA scores <26 or MMSE scores between 19 (17 for 0 years of education) and 26 (24 for 0 years of education), and CDR scores ≤1], and found a positive correlation between MBI and screening-positive cognitive outcomes. It is important to note that these screening tools alone are not sufficient for a definitive diagnosis of MCI or mild dementia, which requires comprehensive neuropsychological, neurological, and etiological evaluation.

As a relatively novel concept, MBI has only gradually been recognized in recent years. MBI is a clinical marker for various neurodegenerative diseases and can be used for diagnosis in the prodromal phase of disease (Dodich et al., 2016). Regarding impaired cognition, MBI may help to identify signs of dementia before the onset of overt screen-positive CI (Yoon et al., 2019). This study employed the MBI-C to assess MBI. Developed by the International Society to Advance Alzheimer's Research and Treatment–Alzheimer's Association in 2017, the MBI-C is a validated tool for diagnosing MBI. Prior to its development, behavioral impairment was typically evaluated using the Neuropsychiatric Inventory (NPI), with patient impairment estimated by mapping NPI items onto MBI domains (Mortby et al., 2018). However, the NPI's short reference period of 1 month increases the risk of false positives. In contrast, the MBI-C requires symptoms to reflect a change in long-term behavior lasting at least 6 months, thereby reducing false-positive cases related to transient symptoms, life stressors, or other medical issues (Lussier et al., 2020). Future research should investigate whether early MBI predicts the rate and extent of cognitive decline in patients with aCSVD. WMHs represent a significant risk factor for late-life screen-positive CI, and in affected individuals, they can be associated with declines in cognitive functions such as processing speed and episodic memory (Jiménez-Balado et al., 2022). Consistently, the present study found that an HWMH burden was associated with more severe screening-positive outcomes than a LWMH burden in aCSVD patients, with significant associations observed for attention, executive function, memory, and orientation. Studies have mainly concentrated on the relationship between WMH and CI (Ali et al., 2023; Crockett et al., 2021), and relatively few studies have explored the link between WMH and MBI. Miao et al. (2021) demonstrated that global MBI status, rather than specific MBI domains, was correlated with a greater WMH volume. Yang et al. (2022) similarly found that patients with larger WMH volumes had higher MBI-C scores. The findings of the present study align with those of the abovementioned studies, indicating that HWMH is associated with more severe MBI than LWMH. Additionally, aCSVD patients with MBI exhibited a greater WMH involvement in the occipital lobes and subcortical structures, rather than in the frontal, temporal, or parietal lobes, compared with those without MBI. This study also demonstrated that MBI could moderate the association between WMH and late-life screen-positive CI, suggesting that comorbid WMH and MBI may exacerbate cognitive decline in aCSVD patients.

### 4.1 Potential neural mechanisms underlying the moderating effect of MBI

The moderating role of MBI in the WMH-cognition relationship may be mediated by disruptions in key neural circuits, particularly the frontal-subcortical pathways. Frontal-subcortical circuits are critical for regulating both cognitive functions (e.g., executive function, attention, memory) and behavioral processes (e.g., motivation, emotion, impulse control; Dodich et al., 2016; Yoon et al., 2019). WMHs, especially those in the subcortical structures and occipital lobes (as found in the MBI group), can disrupt white matter tracts in these circuits, leading to impaired communication between the frontal cortex and subcortical regions (e.g., basal ganglia, thalamus). MBI, characterized by behavioral changes, such as apathy and affective dysregulation, may further exacerbate these circuit disruptions by altering the functional integrity of frontal-subcortical networks. For instance, apathy (a core feature of the impaired drive/motivation domain) is associated with hypoactivity in the prefrontal cortex and striatum, which are key components of the reward and motivation pathway (Miao et al., 2021). When combined with WMH-related structural damage, this hypoactivity may lead to a synergistic decline in cognitive functions that rely on frontal-subcortical interactions, such as executive function and working memory. Similarly, affective/emotional dysregulation may reflect dysfunction in the amygdala-prefrontal circuit, which, when compounded by WMH-induced tract damage, can impair cognitive control and information processing speed.

### 4.2 Specific MBI subdomains contributing to cognitive decline

The results indicated that the impaired drive/motivation and affective/emotional dysregulation subdomains were significantly associated with higher WMH burden (Table 3) and could be the key contributors to the moderating effect of MBI. The impaired drive/motivation subdomain (OR = 1.51, 95% CI: 1.11–2.06, *p* = 0.008) and affective/emotional dysregulation subdomain (OR = 1.55, 95% CI: 1.16–2.08, *p* = 0.003) were independently correlated with HWMH, demonstrating that these behavioral symptoms were more closely linked to WMH-related brain damage. Clinically, apathy (a hallmark of impaired drive/motivation) has shown to predict cognitive decline in various neurodegenerative diseases, including aCSVD (Yang et al., 2022). Apathy may reduce patients' engagement in cognitive activities, leading to disuse atrophy and further cognitive deterioration, particularly when combined with WMH-induced structural damage. Affective/emotional dysregulation, such as low mood and anxiety, may also contribute to cognitive decline by increasing psychological stress, which can impair hippocampal function and exacerbate neuroinflammation (van Straaten et al., 2006). In contrast, other MBI subdomains (e.g., impulse dyscontrol, social inappropriateness) were not significantly associated with WMH burden or screen-positive CI in the present study, suggesting that their role in the WMH-cognition relationship may be less prominent. Future studies should further explore the specific contributions of individual MBI subdomains to cognitive decline in aCSVD, which may inform targeted intervention strategies.

Regarding the small β-values in the moderation models (β = −0.10 for MoCA; β = −0.06 for MMSE), it is important to note that while the statistical significance indicates a meaningful moderating effect, the magnitude of the effect is relatively modest. This may be attributed to the complex, multifactorial nature of cognitive decline in aCSVD, where WMHs and MBI interact with other factors (e.g., vascular risk factors, neurodegeneration) to influence cognition. Clinically, even small moderating effects can be meaningful, as they indicate that identifying MBI in aCSVD patients with WMHs may help stratify individuals who are at a higher risk of cognitive decline. For example, a β-value of −0.10 indicates that for each unit increase in Fazekas scores (WMH burden), the MoCA score decreases by an additional 0.10 unit in patients with MBI compared to those without MBI. Over time, this cumulative effect may contribute to a more rapid progression of screen-positive CI, highlighting the clinical relevance of early MBI detection and monitoring.

This research has several limitations. Firstly, the cross-sectional design of this study could inherently limit causal inference, as it did not allow for the establishment of the temporal sequence among WMH progression, MBI onset, and cognitive decline. Large-scale, prospective longitudinal studies are urgently required to clarify the causal relationships among these factors and validate the predictive value of MBI for cognitive deterioration in aCSVD patients. Secondly, single-center recruitment might introduce selection bias, as the study population was derived from a single hospital's neurology department, which might not fully represent the broader aCSVD patient population. Thirdly, the overall sample size (*n* = 229) is relatively small, particularly for subgroup analyses (e.g., MBI vs. non-MBI comparisons, *n* = 92/137), which might reduce statistical power and increase the risk of type II errors. Future studies with larger, multicenter cohorts are essential to replicate the findings and enhance their generalizability. Thirdly, certain confounding factors could not be fully excluded, such as the absence of AD exclusion using AD-specific biomarkers, subclinical psychiatric symptoms or history (e.g., anxiety, personality traits), and the influence of other imaging factors beyond WMH that might contribute to cognitive deficits in aCSVD. Furthermore, the lack of FDR correction for multiple comparisons might increase the risk of Type I errors, especially for borderline significant results. Therefore, the findings should be interpreted as preliminary, and confirmation in future studies with appropriate correction for multiple comparisons is warranted. Fourth, although we acknowledged the potential for Type I error inflation due to the lack of false discovery rate (FDR) or Bonferroni correction for multiple comparisons in the statistical analysis, we further emphasize this as a key limitation. The exploratory nature of the study and high inter-correlation among cognitive domains led to the decision to report uncorrected *p*-values, but readers should interpret the significant findings with caution, as some associations may be attributable to chance. Future confirmatory studies should apply stricter correction methods to enhance statistical rigor. Fifth, although both the MoCA and MMSE assess overlapping cognitive domains, this study did not determine which is more appropriate for screening screen-positive CI related to CSVD; therefore, cognitive outcomes were synthesized in both scales. Despite this limitation, the simultaneous assessment of all variables enabled us to examine associations at a single time point, which is appropriate for exploratory moderation analyses. Nevertheless, future prospective studies are needed to establish temporal relationships and validate these findings in larger and more diverse populations. Finally, the extent of WMH was evaluated via the Fazekas scale. Future studies should integrate the age-related white matter change scale (van Straaten et al., 2006) and exploit advances in imaging techniques, utilizing diffusion tensor imaging or functional MRI to analyze the specific regions associated with dysfunction and functional connectivity (Duering et al., 2018; Li et al., 2016; Reijmer et al., 2015), as well as using automatic segmentation to accurately and rapidly quantify the burden and progression of WMHs (Li et al., 2020).

## 5 Conclusions

The results indicated that MBI exerted a moderating influence on the association between WMHs and screen-positive CI in patients with CSVD. Further attention should be given to the cognitive function of aCSVD patients with WMH and MBI to prevent rapid progression of the disease. Future studies should validate these findings in cohorts with larger sample sizes, and longitudinal studies are advantageous to determine whether MBI can serve as an early predictor of the rate and extent of screen-positive CI in aCSVD.

## Data Availability

The original contributions presented in the study are included in the article/supplementary material, further inquiries can be directed to the corresponding author.
